# Reimagining Community Mental Health Care Services: Case Study of a Need Based Biopsychosocial Response Initiated During Pandemic

**DOI:** 10.3389/fpsyt.2021.731321

**Published:** 2021-10-07

**Authors:** Poornima Sunder, Anu Sonia Vincent, Meenu K. Saju, Anu S. Moorthy, Godson Paulose, Roshni Robins, Anupama V. Prabhu, M. Arun, Anita Rajah, Chitra Venkateswaran

**Affiliations:** ^1^Mehac Foundation, Kochi, India; ^2^Department of Psychiatry, Believers Church Medical College Hospital, Tiruvalla, India

**Keywords:** COVID 19 pandemic, community mental health, psychosocial support strategies, role of NGO, Mehac foundation, community based organization

## Abstract

Community mental health systems worldwide have undergone transformation in order to accommodate enormous demands of the pandemic and its mitigation efforts. The pandemic created unprecedented challenges that required Mehac Foundation (further referred as Mehac), a not for profit organization based in Kerala, to reassess our care delivery model. The aim of this report is to present a flexible, need-based biopsychosocial response; a case study effectuated by the Non-Governmental Organization (NGO) with a focus on minimizing the impact of COVID 19 on vulnerable communities, while adhering to timely regulations issued by the government. The key aspect of our biopsychosocial response was implementation of a phased approach that was rooted in real time need identification. The strategies will be described under broad headings of (i) adaptations for maintaining continuity of care, (ii) identifying vulnerable subgroups and need based psychological response, (iii) exploring social dimensions of the pandemic and implementing strategies to address them, (iv) ensuring team well-being and enhancing skills to effectively respond to the challenges.

## Introduction

COVID 19 pandemic has disrupted the functioning of mental healthcare worldwide and has presented a dreadful challenge to community mental health care (1). It has without doubt resulted in an instant escalation of mental health issues globally. At the beginning of the pandemic, there were predictions about India's inability to cope “*India has a high risk of community transmission because of crowded living conditions, congested cities, a large slum dwelling population, poor health-care facilities, low educational attainment, and high levels of poverty*” (2). In addition, there have been concerns about mental health and psycho-social consequences of self-isolation and sudden lockdown, which has affected usual life and routines of people and in turn has led to an increase in loneliness, anxiety, depression, insomnia, substance use, self-harm, or suicidal behavior (3, 4). The psychosocial impact of the pandemic still remains mostly unresolved and unaddressed; a recent survey by the Indian Psychiatric Society shows that 2/5th of the people surveyed were experiencing common mental health disorders since the coronavirus outbreak (3). Managing the direct and secondary effects of the pandemic and also implementing the solutions are difficult for developing countries like India, with an approximate population of over 1.35 billion people.

As per the National Mental health Survey (NMHS-2016), nearly 150 million Indians (urban > rural) are in need of active mental health interventions, but the treatment gap for overall mental morbidity was 84.5% (5). Though Kerala fares better in terms of number of personnel and health facilities, it has a higher suicide rate of 24.3 when compared to national average of 10.4. India has a three-tiered health-care system for delivering preventive and curative services along with private health care facilities (6). District mental health programme forms the fulcrum of service delivery by the government for mental illness at primary care level however, there are bottlenecks at various levels from policy to implementation and utilization (7). Hence, to bridge the existing treatment gap and address inherent disparities in the system, scaling up of services with alternatives like Non-Governmental Organization (NGO)'s are invaluable in Indian setting. This scenario was made more evident during the ongoing pandemic where maintaining services at grassroot levels were further strained.

## Mental Health Care and Research Foundation Model

Mental Health Care and Research Foundation (Mehac) Foundation, is a not for profit organization working in the southern part of Kerala, India, since the year 2008. Pain and palliative care (PPC) movement (8) in Kerala laid the foundation for Mehac to eventually adapt palliative care principles to mental health care. The mission of the foundation is to evolve and propagate a new model of mental health care, by strengthening existing systems in the community and thereby increasing community participation. Mehac executes a flexible model of community psychiatric care through its partnership with community-based organizations (CBO) based on Public-Private-People Partnership (4P's). Collaborative arrangements with Panchayats (local self-government department), private sector, and civil society help Mehac to sustain programmes in the community and cater to the marginalized sections of the society. Over the past 12 years, the foundation has touched the lives of more than 5,000 people with mental health issues in six districts of Kerala delivered by a multidisciplinary team.

Mehac emphasizes cost effective quality care; it had humble beginnings with donations from likeminded philanthropists. Currently the services are supported by governmental organizations like Panchayats, partnering civil society organizations and individual donors. Corporate organizations have come forward with their social responsibility grants as well.

Mehac's volunteer system was developed based on the Neighborhood Network in Palliative Care initiative (NNPC) which is an exemplar model for resource poor settings (9). A volunteer in the community would be someone willing to contribute specific time for the care of people, who undergoes training and is willing to be supervised, and works with a team. Most of them would have passed their high school education. They vary from lay volunteers to community level health workers like Accredited Social Health Activist (ASHA) (10). They are trained and empowered to address stigma and facilitate early identification and intervention through referral pathways. These volunteers are identified by local CBOs from within their communities and they act as a bridge between the expert team and community. In a collectivistic society like India, the family of a Person with Mental Illness (PwMI) plays a major role in caregiving (11). Mehac considers Family as the unit of care and ensures their maximal empowerment to increase participation in management of our patients. Engaging through volunteers and families has ensured high rates of compliance and prompt identification of relapses.

Mehac functions by empowering CBOs to take ownership by providing an expert consultancy role and a stepped care approach enabling delivery of medical, psychological, and rehabilitation services. Access to free medicines and necessary infrastructure support are ensured by the partners. We run a total of 22 clinics, 68% of which function in collaboration with the local NGOs and 32% in partnership with Panchayats. Prior to the pandemic, consultations were done for 2,852 patients through clinics (88%) or home visits (12%) requiring travel (on an average about 50–60 km per day). Medications were also dispensed either directly at clinics (92%) or through home visits (8%).

## National and State Response to Pandemic

India's first COVID patient was reported from Kerala in January 2020, which was contained with active measures (4). However, by February 3^rd^ Kerala, had declared a state calamity and by March 11^th^ WHO, had declared it a pandemic. The situation worsened and by March 22^nd^ “Janata Curfew” a one day voluntary lockdown was announced. On March 24^th^ Kerala declared a statewide lockdown, followed by Indian Government imposing a nationwide lockdown on 25^th^ March. India's lockdown was extended till May 31^st^, it was one of the strictest and longest in the world (4). The nationwide lockdown was carried out in four phases; the initial two phases were very strict with restricted movements across the country, and the latter two phases saw gradual and contextual relaxations based on case load with clearly defined containment zones. By June 8^th^, phased reopening was announced. The lockdown, inspite of its perceived success, also drew wide attention because of its negative socioeconomic impact (12). Kerala government, by pooling existing facilities in the government, private and voluntary sector was able to implement innovative strategies, effectively cutting down the spread of COVID 19, and minimizing its psychosocial impact during early days of the pandemic (13, 14).

## Biopsychoscoial Response Model Implemented by Mehac

**Figure 1:**
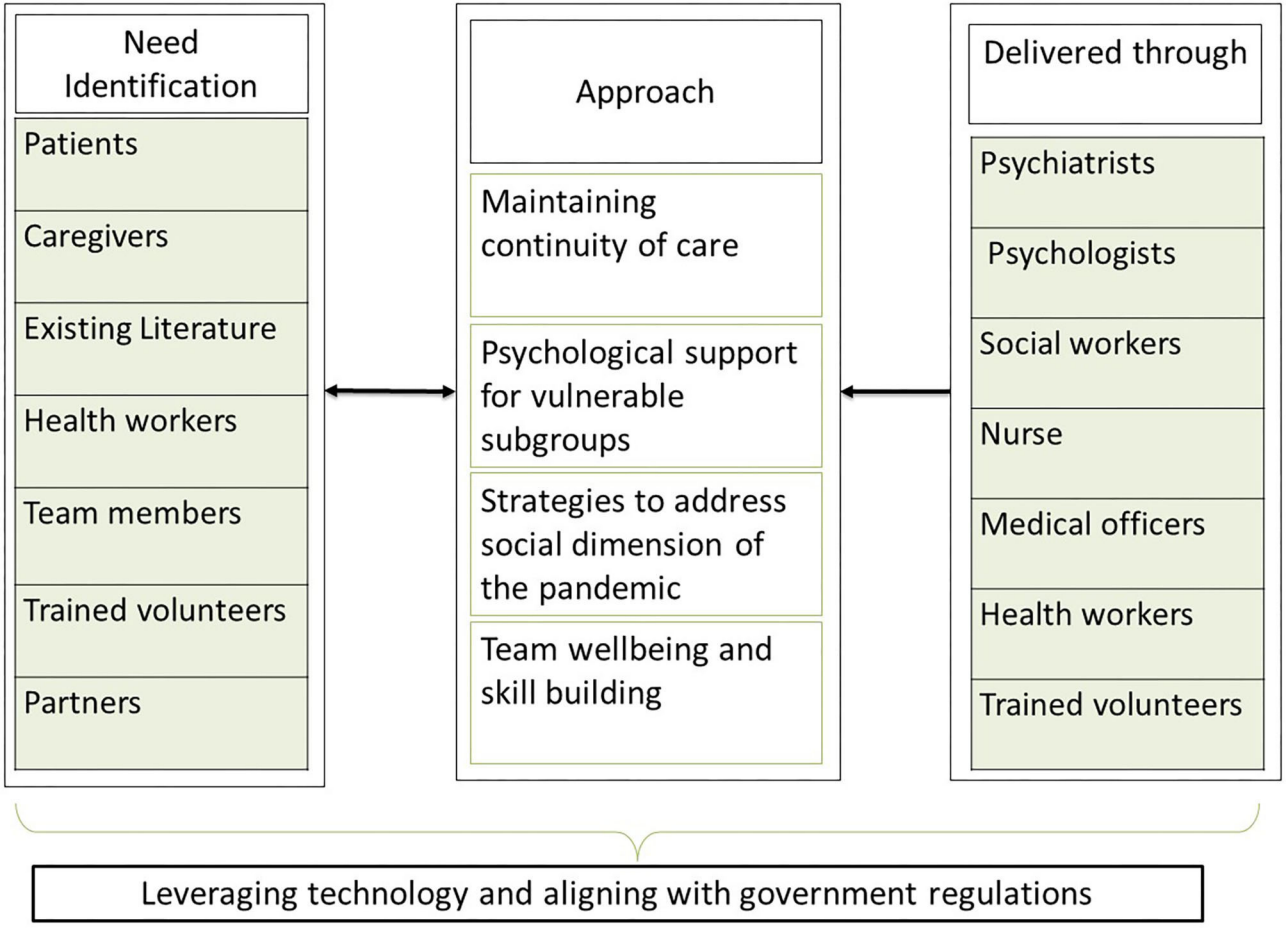
Phases of need based Biopsychosocial response model implemented by Mehac, Kerala, India based on the COVID 19 pandemic.

The overall functioning of our CBOs were adversely affected by the sudden lockdown. In response, we adopted a multipronged-multidisciplinary biopsychosocial approach (15) (see Figure 1), which was based on evidence from existing literature and real time need identification through continuous feedback from patients, their families, community volunteers, community health workers, community partners, and the team members. More than 90% of our beneficiaries belong to the lower socioeconomic status (16) with limited access to mental health services, hence providing consistent treatment services to them was crucial. This was achieved by restructuring our service delivery with adaptations to existing biopsychosocial approaches and effectively leveraging available and affordable technology options. The strategies were devised giving careful attention to the social, cultural, and economic background of the beneficiaries as well as the updated regulations by the Government.

The key aspect of our Biopsychosocial response was time sensitive adaptation to the needs arising in the community. The strategies were implemented in a phased manner: (i) initiation-phase from the time the pandemic was declared a state calamity to the first two phases of strict lockdown (ii) the transition-phase from third phase of Lockdown to stepped reopening and (iii) the continuation-phase from reopening to new normal functioning.

**Figure 2:**
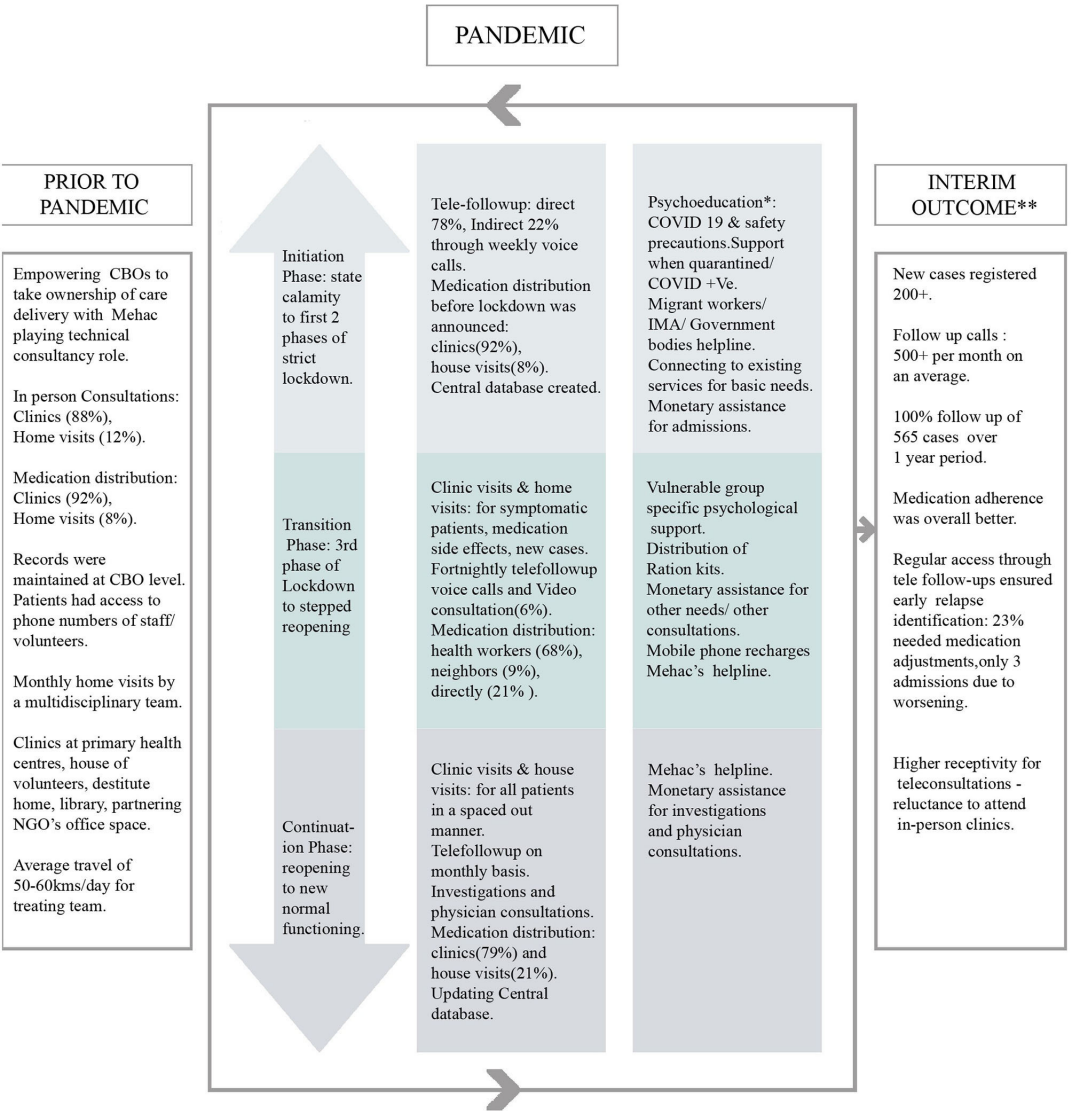
Biopsychosocial response model implemented by Mehac, Kerala, India in response to COVID 19 pandemic.

We adopted a need based phased response not only to follow governmental guidelines but also to be prepared for future resurgence (17). The strategies described in a phased manner are flexible and cyclical and not linear (see Figure 2). It will be described under broad headings of (a) adaptations for maintaining continuity of care, (b) identifying vulnerable subgroups and need based psychological response, (c) exploring social dimensions of the pandemic and implementing strategies to address them, and (d) ensuring team well-being and enhancing skills to effectively respond to the challenges.

## Adaptations for Maintaining Continuity of Care

COVID 19 made restructuring of our services mandatory. Around 95% of our patients suffer from severe mental disorders and hence maintaining continuity of care during the pandemic was crucial. Out of a total number of 2,852 patients in our service, 565 had regular direct followup (teleconsultations/visits), for rest of the stable patients from referrals and outreach centers, more than 500 needs-based help was extended.

During the initiation phase, the team tried to maintain regular follow-up with patients and their families either directly (78%) or indirectly (22%) through volunteers, health workers, or neighbors. Follow up calls were done mainly through voice calls as that was the easily available option to overcome existing digital divide (18). Initial follow-ups were done on a weekly basis, wherein enquiry was made regarding current status of patient, their biological and socio occupational functioning as well as medication adherence. As there were predictions of increased chances of relapse or worsening in patients with pre-existing mental health issues as was being reported from the world over (19–21), these strategies helped in identifying early signs of relapse. In the initiation phase, patients were followed on a weekly basis and then it was eased to fortnightly during the transition phase as most of the patients were stable. In the transition phase, we conducted a survey to understand if patients preferred returning to in-person clinics. Sixty-three percent wanted to visit clinics whereas 37% preferred home care visits.

“*COVID cases are increasing here, all of you stay at home. R (volunteer with Mehac) ensures that medicines reach us on time you call and ask about me and my daughter, do not come to hospital to see us now. Ask everybody else in the team to stay safe, we will call promptly if we have any other problems*.”

- Mother of a 20 year old PwMI

Mehac followed a hybrid model further into the transition phase; incorporating home visits, in-person clinics, and regular tele follow ups. Initially patients who needed in-person consultation were seen selectively. An arbitrary criteria was applied and those who were symptomatic with medications changes being indicated, or those who were experiencing any side effects of medications were seen in nearby clinics with well-spaced out appointments to ensure social distancing and to avoid overcrowding. Each of the appointments were fixed with reiteration of safety precautions, as patients with severe mental illness were predicted to have difficulty following the same in view of impaired insight and decision making capacity (14). Strategies were constantly revised and we shifted toward virtual clinics wherein the social workers or psychologists would follow up with the patient along with a psychiatrist. Video consultations were arranged for 6% of patients, with help of younger family members or neighbors. As the situation eased, we encouraged patients and their family members to come to clinics along with continuation of tele follow up during the interval between clinic visits. In-person consultations were arranged only when need arose for the same. After the lockdown was lifted, tele follow ups continued with adjusted timings, as many of our patients and family members became unavailable during working hours.

All emergency consultations were either seen in-person or through virtual means. Existing ties with governmental and other volunteering agencies enabled Mehac to access local health care professionals and ensure timely help, thereby minimizing need for admissions. Medicine dosage was altered for those patients who couldn't be given long acting depot injections and they were monitored closely for early signs of relapse. Patients on clozapine were also closely monitored for any side effects and relapse of symptoms.

Mehac tried to address the issues of patients with comorbidities since patients with severe mental illness generally have higher rates of comorbidities and are vulnerable due to substantial disparities in access to health care (22). COVID 19 further compounded this vulnerability. Around 96 persons were identified with co morbid conditions like diabetes, hypertension, parkinsons disease, dementia, epilepsy, stroke, paraplegia cardiovascular disorders, chronic respiratory illnesses, chronic renal failure, etc. Home visits and video consultations were conducted exclusively for them in order to minimize risk of contagion. For similar concerns, family members with comorbidities, especially those that were elderly and were the sole caregiver for patients were also discouraged from visiting in-person clinics. In a survey conducted, we found people with comorbid conditions were hesitant to access health care services and mean duration from last physician visit was 6 months. Mehac utilized its ties with government and local volunteering organizations to link such patients to existing services for investigations and consultation.

“*This is the first time in my life that I have not been able to find a job for such a long duration, it's all because of the pandemic. Government ration kits and the free medicines you provide are keeping my family from starving. My wife has illness since 20 years, before a lot of my earning was spent on medicines and hospital bills as she had relapses when I could not afford medicines. I can't imagine how we would have got through these tough times without these medicines reaching us so promptly.”*

- Husband of a 50 year old PwMI

Challenges in maintaining a medication supply was reported worldwide (1, 23). Continuing the same without any disruption in the absence of our established outpatient set up was another major obstacle that we faced from the early days of pandemic. To ensure adequate supply through the period of lockdown, we communicated in advance with more than 90% of our patients to collect medicines. Adequate stock of medications was ensured by coordinating with various stakeholders, ranging from medicine suppliers to local partners and volunteers. Mehac raised funds and supplemented purchase of medications when the shortage was due to administrative/financial reasons. We created various subzones within the zones of our existing clinic areas and when patients were not able to travel to clinics, we coordinated with community volunteers, health workers, or neighbors to ensure seamless distribution of medications. When needed, field supervisors stepped up to make arrangements and ensured inter organizational cooperation between local partners. Timely improvisations were made to our existing model with 68% of medicines being distributed through help of health workers, 9% through neighbors, and 23% being collected directly from clinics. Even when lockdown was eased and eventually lifted, we continued a similar mode of delivery of medications in coordination with various stakeholders demonstrating long term learning. Overall these adaptations ensure 100% follow up of our patients with improved compliance to medications.

## Identifying Vulnerable Subgroups and Psychological Response

When it comes to COVID 19, certain groups were found to be more vulnerable psychologically. Among them were persons with severe mental illness, elderly people, children (especially those with mental health issues) and migrant workers (24–30). Kerala's response in providing psychosocial support for these groups has received worldwide recognition. Telemedicine portal e-sanjeevani for teleconsultation across the State and “Ottakalla oppamundu” (You are not alone, We are with you) for providing psychosocial support were a few of the models recognized by the WHO. The government adopted an inclusive approach and addressed the special needs of vulnerable population (13, 14, 31).

“*I can't talk to anybody about my worries, my daughter doesn't support me and she has no time to listen to my problems even if I call her. I am not able to go out like before and nobody visits me now. You call and enquire about me regularly; you are more like a family to me now. Though you can't come, you made sure that help reached me through ASHA worker. Knowing that I can call you gives me hope.”*

- 69 Year old PWMI, living alone

Incorporating these principles, Mehac partnered with government bodies and other local NGOs to provide psychosocial support to vulnerable populations. In partnership with the government bodies, Mehac was involved in providing teleconsultation services to migrant workers and COVID positive patients. Additionally, partnering with IMA's (Indian Medical Association) telemedicine services, Mehac extended support for people who were COVID positive and in quarantine. Apart from this, for its existing patients Mehac provided weekly teleconsultation services especially to people with severe mental illness and their caretakers, elderly, children with mental health issues, people living alone, those with co morbidities and those who tested positive for COVID. In the initial phase, we provided psychoeducation regarding COVID symptoms, precautionary measures and its impact. Throughout the period of lockdown special attention was also given to families of PwMI and their distress was addressed. In our experience, families were resilient and their concerns were more related to meeting financial needs. Though there were media reports of increased violence during lock down, among our population we encountered one such incident and were able to liase for help with the established governmental women helpline. Psychological first aid principles were applied in practice when indicated to allay fears. Supportive psychotherapy was also provided on an individual basis when needed. Both physical and mental health issues of these groups were addressed by partnering with available resource people within the community.

## Exploring Social Dimensions of the Pandemic and Implementing Strategies to Address Them

“*My husband and me have not been able to find any jobs as nobody is hiring daily wage workers now. We were able to manage somehow though the Government ration kits, but the debts in the grocery shop were piling up and we couldn't buy vegetables or meat for our children. With the money you gave me instead of the ration kit, I could pay my debts at the shop and buy fish for my children after a long time. I can't explain how happy my children were.”*

- Wife of 56 Year old PwMI

COVID 19 has brought a complete breakdown of social support systems across the world. In addition, people with existing mental, neurological and substance use disorders constitute an already economically vulnerable group who are susceptible to chronic poverty and relapse (31). The Kerala government initiated timely measures such as ensuring food kits to all, supplying mid-day meals to children at home, ensuring free meals for migrant workers etc., to mitigate psychosocial impact of the pandemic (32, 33). During the initiation phase, follow-up by the team revealed that most of the ground level apprehensions were related to interruption in the supply of basic material needs due to the lockdown. This was addressed by connecting them to various services offered by the government, voluntary organizations, or other NGOs. After lockdown was lifted, the government continued supplying ration kits and other basic necessities, but people lacking ration cards or those wrongly categorized as Above Poverty Line (APL) (34, 35) had difficulty in availing these services. Hence, Mehac stepped up and identified such people in each clinic and started distributing ration kits (*n* = 31) and other provisions as needed. Even though the majority of people had access to food through various schemes, many of them were seen struggling to meet other basic needs as they didn't have sufficient money at hand due to job loss and unemployment. Monetary assistance was provided for such people. Mehac also took care of consultation fee and transport assistance for those who warranted hospital visits for other physical issues or to facilitate admissions when symptomatic.

Research shows that maintaining social support has higher scores on recovery for people with severe mental illness (36). The pandemic along with its mitigation efforts have resulted in making telecommunication services a basic necessity. Hence, maintaining regular connectivity with our beneficiaries and helping them to connect with their dear ones was vital in order to keep them calm and informed. Identifying this need, Mehac made efforts to recharge mobile phones of beneficiaries when needed. Realizing the magnitude of crisis on the ground Mehac conducted timely fundraising through individual donors and corporations who were willing to support through their social responsibility grants.

## Ensuring Team Well-Being and Enhancing Skills to Effectively Respond to the Challenges

“*How was I going to understand people emotions through a voice call? I rely so much on talking face to face to do my work in the community. I was apprehensive about this however, discussions with my team mates and exercises like role plays gave me the initial confidence. My patients and their family helped me gain more insights through the sessions over phone. After initial few calls I realized that, though my barriers increased in reaching out to them in person, they could reach out to me better, which in turn meant very few of my patients had any major problems.”*

- Social worker with Mehac

Healthcare workers are considered a vulnerable group due to high risk of infection, increased work stress, and fear of spreading the infection to their families (37, 38). In order to cope with challenges that the pandemic posed, it was crucial to strengthen team dynamics and foster cooperation. Peer group surveys identified stress of the ongoing pandemic and guilt of spreading infection to family members prevalent among team members. The remote mode of operating necessitated work from home which resulted in team members facing problems within their personal sphere as well. However, the existing dynamics and rapport within team members ensured a sense of solidarity that in turn helped to ease apprehension and worries. Strategic adaptations were made through frequent communications and feedback. The team made an effort to identify and gather accurate information about safety precautions and government rules and regulations regarding the pandemic. The information gathered was passed on to the beneficiaries through teleconsultation which helped in dispelling anxiety created by misinformation. The whole process of acquiring and disseminating accurate information helped to reduce the team's anxiety as well.

Regular discussions helped troubleshoot problems and refine strategies. Different communication channels were established and various contextual groups were created to develop strategies and to attend to emergencies. Even though there were frequent connectivity issues, the team experimented with various online platforms such as Zoom, Google Meet, etc., to identify a platform that was comfortable for all. When gaps in patient follow up were identified, the team shifted to password protected excel spreadsheets for ease of follow up and communication. Enhancing skills like planning and communication was identified as a felt need among the team members. Various training sessions with specific modules, empowerment, and review sessions were conducted. The team actively participated in online webinars to update knowledge regarding COVID 19 and its impact as well as psychosocial strategies to address it. Team members were part of developing a “Resource Toolkit for Low and Middle Income Countries for Palliative Care in COVID 19” (39) which was also extended as an online training program.

## Conclusion

The Mehac model of task sharing (40) and community participation provided the advantage of a strong ground support system that helped in an expeditious transition to a remote mode of operating. The readaptations made by leveraging technology enabled us to strengthen our care delivery model further; ensuring overall better follow up with improved compliance to medication when compared to previous year. A phased approach rooted in real time identification of perceived needs and barriers enabled us to not only sustain continuity of care but also to focus on social needs. These strategies in turn helped in building a sense of community support and seem to enhanced resilience in our vulnerable population (41). Constant evolution of the model based on government regulations and identified needs helped in designing localized solutions with maximal use of available community resources, thereby encouraging our beneficiaries to exercise agency. Responsive leadership and open channels of communication within the team helped in achieving better patient care and ensuring team well-being.

The abruptness and evolving nature of the pandemic resulted in initial difficulty in devising systems for tracking data, hence the results mentioned here are interim, impressionistic, and clinically based. Limitations include not gathering quantitative and experiential data to measure outcomes; there was no formal evaluation during the pandemic. Technology was not leveraged enough to conduct support groups, caregiver sessions for families, or awareness programs which were conducted on a regular basis prior to the pandemic. For future pandemics or other community crisis, ideally we will be prepared to assess the outcomes of this model implemented during the COVID 19 pandemic using mixed methods with quantitative tools and surveys to measure outcomes as well as qualitative evaluation to study the process indicators like satisfaction, readiness, challenges, and barriers along with acceptability, appropriateness, adoption, and feasibility of interventions through key informant interviews and focus groups. Replication requires testing adaptations in different societal structures. Generating discussions around political will and societal attitudes are crucial to designing culture- and context-specific interventions as well as pathways for funding.

The inherent ability to adapt to changes, while respecting individual autonomy and empowered participation of various stakeholders are crucial for a sustainable community model. Sensible local application of broader concepts of cost effective biopsychosocial approaches mentioned here can ensure accessibility of care for even the most underserved sections of the society in a resource limited setting. Sharing experiences and evaluations, negotiation with policy makers are important components to replicate resilient community led approaches in other settings.

## Data Availability Statement

The raw data supporting the conclusions of this article will be made available by the authors, without undue reservation.

## Author Contributions

PS and AV were major contributors to conception, designing, and writing up the manuscript. MS, AM, GP, and AP carried out surveys, helped in updating database, and wrote sections of the manuscript. RR helped with interpreting database and carried out proof reading. MA, AR, and CV gave valuable inputs to improve the overall outlook. All authors contributed to manuscript revision, read, and approved the submitted version.

## Conflict of Interest

The authors declare that the research was conducted in the absence of any commercial or financial relationships that could be construed as a potential conflict of interest.

## Publisher's Note

All claims expressed in this article are solely those of the authors and do not necessarily represent those of their affiliated organizations, or those of the publisher, the editors and the reviewers. Any product that may be evaluated in this article, or claim that may be made by its manufacturer, is not guaranteed or endorsed by the publisher.
